# Attitudes and Behavior towards Interprofessional Collaboration among Healthcare Professionals in a Large Academic Medical Center

**DOI:** 10.3390/healthcare8030323

**Published:** 2020-09-06

**Authors:** Benjamin E. Ansa, Sunitha Zechariah, Amy M. Gates, Stephanie W. Johnson, Vahé Heboyan, Gianluca De Leo

**Affiliations:** 1 Applied Health Sciences Program, College of Allied Health Sciences, Augusta University, Augusta, GA 30912, USA; szechariah@augusta.edu (S.Z.); amgates@augusta.edu (A.M.G.); sjohnson8@augusta.edu (S.W.J.); 2 Institute of Public and Preventive Health, Augusta University, Augusta, GA 30912, USA; 3 Morrison Healthcare, Sandy Springs, GA 30350, USA; 4 Neonatal Intensive Care Unit, Augusta University Health, Augusta, GA 30912, USA; 5 Occupational Therapy, College of Allied Health Sciences, Augusta University, Augusta, GA 30912, USA; 6 Health Economics and Modeling Division, Population Health Sciences Department, Medical College of Georgia, Augusta University, Augusta, GA 30912, USA; vheboyan@augusta.edu; 7 Department of Interdisciplinary Health Sciences, College of Allied Health Sciences, Augusta University, Augusta, GA 30921, USA

**Keywords:** interprofessional collaboration, attitudes, behavior, healthcare professionals, academic medical center

## Abstract

The increasing rates of comorbidities among patients and the complexity of care have warranted interprofessional collaboration (IPC) as an important component of the healthcare structure. An initial step towards assessing the effectiveness of collaboration requires the exploration of the attitudes and experience of healthcare professionals towards IPC. This online survey aimed to examine the attitudes of healthcare professionals working in a large public academic medical center toward IPC in patient care and the healthcare team, and their behavior and experience regarding IPC. The rankings, according to the perceived importance among the respondents, of the four Interprofessional Education Collaborative (IPEC) core competencies (values/ethics, roles/responsibilities, interprofessional communication, teams/teamwork) were assessed. There were strong but varying levels of consensus among healthcare professionals (*N* = 551) that IPC facilitates efficient patient care, improves patient problem-solving ability, and increases better clinical outcomes for patients. They acknowledged that IPC promotes mutual respect within the healthcare team and providers’ ability to make optimal patient care decisions. However, overall more than 35% of the respondents did not attend multidisciplinary education sessions (grand rounds, seminars, etc.), and about 23% did not participate in bedside patient care rounds. Interprofessional communication was ranked as the most important IPEC core competence. Although the attitude towards IPC among healthcare professionals is strongly positive, many healthcare professionals face challenges in participating in IPC. Institutional policies that facilitate interprofessional learning and interactions for this group of healthcare professionals should be formulated. Online distance learning and interactions, and simulation-enhanced interprofessional education, are options for addressing this barrier. Hospital administrators should facilitate conducive work environments that promote IPC, based on IPEC core competencies, and promote programs that address the challenges of IPC.

## 1. Introduction

Collaboration has been defined as the cooperative arrangement in which two or more parties work together towards completing a task or achieving a common goal [1]. Interprofessional collaboration (IPC) in healthcare is a partnership among diverse health professionals to provide quality care to patients, families and caregivers [2,3]. IPC utilizes both the individual and collective skills and experience of team members, allowing them to function more effectively and deliver a higher level of care than when working alone [4]. Most significantly, patients are more likely to receive the highest quality of care if healthcare professionals understand each other’s roles and appreciate each other’s skills. IPC involves responsibility, accountability, coordination, communication, cooperation, assertiveness, autonomy, and mutual trust and respect among healthcare professionals [5].

The World Health Organization (WHO) framework has provided strategies and models to assist professionals in designing and implementing a team-approach [2], and the Interprofessional Education Collaborative (IPEC) core competency document has helped to frame the national dialogue on the need for interprofessional education and practice as a catalyst for improving team-based patient care and enhancing population health outcomes [6]. IPEC proposed four Core Competencies for successful Interprofessional Collaborative Practice [6]: Values/Ethics, Roles/Responsibilities, Interprofessional Communication, and Teams and Teamwork.

The need for effective teams is paramount due to rising co-morbidities and increasing complexity of patient care [7]. IPC reduces the time and costs of hospitalization, decreases hospitalization and readmission rates, and improves healthcare for older adults with chronic illnesses [8,9,10]. IPC also enhances patients’ satisfaction, reduces the rates of medical errors [10], and improves health outcomes and quality of care [11,12,13,14]. Moreover, IPC facilitates the efficient use of healthcare services by improving the coordination of care, reducing service duplication, and promoting communication among the healthcare team. It also facilitates greater role clarity and enhances job satisfaction and well-being among team members [8,10]. In addition, IPC offers appropriate referral patterns, greater continuity and coordination of care, and better collaborative decision-making with patients [12,14].

An important component of any healthcare structure is the effectiveness of IPC. This may be assessed by evaluating some of its components such as communication and coordination. One study [15] made by the California Academy of Family Physicians found that slightly more than half of primary care physicians were satisfied with their communication with hospitalists. Many of them were dissatisfied with the discharge summaries, which they considered to be too detailed and often arrived late after the patient’s first post-discharge appointment. Untimely communication made it difficult for the primary care physicians to answer patients’ questions and provide adequate care. Results from a published systematic review [16] showed that clinical coordination improves quality of care and saves money.

An initial step towards assessing the effectiveness of collaboration is to explore the attitudes and experience of healthcare professionals towards IPC. Although respondents’ perspectives on interprofessional education (IPE) in educational settings have been extensively examined by several studies [17,18,19,20,21,22,23], understanding healthcare professionals’ perspectives on IPC in specific healthcare settings is paramount to addressing the barriers to effective IPC. The objective of this study was to explore the perspectives of healthcare professionals towards IPC in a large public academic medical center. The specific aims were to evaluate the attitudes of healthcare providers toward IPC regarding patient care and the healthcare team, and to examine the behavior and experience of healthcare professionals regarding IPC, based on their experience in the work environment. The rankings according to the perceived importance among the respondents, of the four IPEC core competencies (values/ethics, roles/responsibilities, interprofessional communication, teams/teamwork) were also assessed.

## 2. Materials and Methods

### 2.1. Survey Design

This cross-sectional study was designed using survey questions from previously validated and published instruments [18,20,24]. The questions were tailored to fit the healthcare work environment, and included empirical, Likert scale and open-ended questions. The survey was designed and administered through the Qualtrics platform (Qualtrics, Provo, UT, USA) and consisted of five sections: (1) respondents’ characteristics, (2) knowledge and attitudes regarding interprofessional education and collaboration, (3) attitudes toward interprofessional collaboration regarding patient care, (4) attitudes toward interprofessional collaboration regarding the healthcare team, and (5) interprofessional collaboration behavior and experience.

The study participants were asked to rank the four core IPEC competencies (Ethics, Communication, Roles/Responsibilities, Teams/Teamwork) in order of importance. Each ranking order could only be assigned once.

### 2.2. Ethical Considerations

Approval for this study was obtained from the institutional review board of the academic medical center being investigated (IRB # 1138392-2), and informed consent was provided by the study participants.

### 2.3. Study Site and Study Population

This was a study of healthcare employees from a large academic medical center in Georgia, United States. The medical center is a public not-for-profit corporation that manages the clinical operations associated with a university. The health system includes a 478-bed medical center, 154-bed children hospital with a level IV Neonatal Intensive Care Unit (NICU), 80 outpatient practice sites, and a critical care center that houses a 13 county regional level 1 trauma center. It employs over 4700 healthcare professionals of varying disciplines.

Participants for this study were recruited through the medical center’s healthcare employee database. A total of 4252 employees comprising of attending physicians, fellows and residents, nurses, nurse practitioners, physician assistants, respiratory therapists, occupational therapists, physical therapists, social workers, registered dietitians, pharmacists, and speech pathologists were invited to participate in the survey. Participants were healthcare employees working in the adult and pediatric areas of the medical center including inpatient, intensive care units, and outpatient clinics. A total of 663 healthcare professionals responded to the survey invitation, and the response rate was 15.6%.

### 2.4. Survey Administration

An initial pilot survey was distributed electronically to 47 hospital employees to clarify any ambiguity with the survey questions. Responses to the survey reflected that the participants were able to comprehend the questions and they did not identify any areas of ambiguity. The finalized anonymous survey was distributed through Qualtrics to the rest of the 4205 participants. Two email reminders were sent after two weeks and four weeks of the initial email in order to encourage participation in the survey. The survey was accessible for six weeks from the initial email distribution. Participation in the survey was voluntary, and informed consent was received from each of the participants. Investigators received de-identified data of the survey responses through Qualtrics.

### 2.5. Statistical Analysis

All data were reviewed for completeness, and incomplete responses were excluded from the study. Healthcare professionals with almost similar roles were combined for the purpose of data analysis. Attending physicians, fellows, and residents were grouped as physicians (MD), while registered nurses and licensed nurses were grouped as nursing (RN). Physician assistant (PA), respiratory therapists (RT), occupational and physical therapists (OT/PT), social workers (SW), registered dietitians (RD), and speech pathologists (SP) were also grouped as allied health professionals (AH). Advanced nurse practitioners (NP) and pharmacists (RPH) were not combined with any other group and were analyzed as separate entities.

Descriptive statistics were employed to analyze respondents’ characteristics, their responses about attitudes toward IPC in patient care and those regarding the healthcare team, and their behavior and experience regarding IPC. Scores were assigned to each of the Likert Scale ratings with a range of ‘1’ to ‘5’. “Strongly disagree” was scored as ‘1’ and “strongly agree” was scored as ‘5’. Chi square test was used to determine the differences between grouped professions. The IPEC core competencies rankings were also analyzed by frequencies. Statistical significance was set at the level of *p* < 0.05. Data were analyzed using SPSS version 25 (IBM SPSS, Inc., Armonk, NY, USA).

## 3. Results

### 3.1. Characteristics of Study Participants

A total of 663 healthcare professionals responded to the survey invitation (response rate = 15.6%). Only surveys completed in their entirety (*N* = 551) were included in the analyses. The majority of respondents were females (*n* = 425, 77.1%), White (*n* = 408, 74%), with a bachelor’s degree as their highest level of education and had more than 20 years of experience (*n* = 235, 42.6%). The ages of the respondents were mostly distributed between 30–59 years (*n* = 413, 75%), and the majority worked in the inpatient department of the medical center (*n* = 291, 52.8%). Most of the respondents were nurses (*n* = 316, 57.4%), while pharmacists (*n* = 14, 2.5%) and advanced nurse practitioners (*n* =15, 2.7%) accounted for the least number of participants (Table 1).

### 3.2. Attitudes toward Interprofessional Collaboration

#### 3.2.1. Attitudes toward Interprofessional Collaboration Regarding Patient Care

The study participants responded to questions about their attitudes towards IPC regarding patient care (Table 2A). A large proportion ‘agreed’ or ‘strongly agreed’ (*n* = 493, 89.5%) that patients receiving interprofessional care are more likely than others to be treated as a whole person. Almost all of the respondents ‘agreed’ or ‘strongly agreed’ (*n* = 546, 99.1%) that patients would ultimately benefit if healthcare professionals worked together to solve patient problems. Most of the respondents ‘agreed’ or ‘strongly agreed’ (*n* = 496, 90%) that “give and take” (compromise) among team members helps providers make better patient care decisions. Respondents also ‘agreed’ or ‘strongly agreed’ (*n* = 477, 86.6%) that an interprofessional approach makes the delivery of patient care more efficient, and they ‘agreed’ or ‘strongly agreed’ (*n* = 509, 92.4%) that reporting clinical observations to a multidisciplinary team helps the team members to better understand the role of other healthcare professionals. Similarly, 94.9% (*n* = 523) ‘agreed’ or ‘strongly agreed’ that IPC increases the ability of the healthcare team to understand clinical problems. Although the majority (*n* = 369, 67%) ‘disagreed’ or ‘strongly disagreed’ that working in an interprofessional manner unnecessarily complicates patient care, 10.7% (*n* = 59) remained neutral while 22.3% (*n* = 123) ‘agreed’ or ‘strongly agreed’.

#### 3.2.2. Attitudes toward Interprofessional Collaboration Regarding Healthcare Team

Table 2B shows the attitudes towards IPC regarding the healthcare team. A majority of the respondents ‘agreed’ or ‘strongly agreed’ (*n* = 458, 83.1%) that IPC helps healthcare professionals think positively about the healthcare team. In addition, respondents ‘agreed’ or ‘strongly agreed’ (*n* = 542, 98.4%) that it is of critical importance for the healthcare team to have effective communication skills for improved patient outcomes. A majority of respondents ‘agreed’ or ‘strongly agreed’ (*n* = 432, 78.4%) that IPC allows healthcare professionals to understand their role limitations, and they also ‘agreed’ or ‘strongly agreed’ (*n* = 540, 98%) that, for IPC to be effective, the healthcare team needs to trust and respect each other.

Respondents ‘agreed’ or ‘strongly agreed’ (*n* = 503, 91.3%) that team meetings foster communication among members from different professions and disciplines, and that working in an interprofessional environment keeps healthcare professionals enthusiastic and interested in their jobs (*n* = 410, 74.4%). Slightly more than half of the respondents ‘agreed’ or ‘strongly agreed’ (*n* = 301, 54.6%) that working in an interprofessional manner requires additional time. On the other hand, 19.4% respondents (*n* = 107) ‘disagreed’ or ‘strongly disagreed’ while 26% (*n* = 143) remained neutral. A majority of respondents ‘agreed’ or ‘strongly agreed’ (*n* = 487, 88.4%) that, for IPC to be effective, members of the healthcare team must work within their scope of practice.

### 3.3. Interprofessional Collaboration Behavior and Experience

In the survey, the study participants were asked about their interprofessional collaboration behavior and experience (Table 3). Most of the respondents ‘agreed’ or ‘strongly agreed’ (*n* = 433, 81.3%) that they are included in patient care decision making. Similarly, a majority of them ‘agreed’ or ‘strongly agreed’ (*n* = 297, 64%) that they participate in multi-disciplinary patient care rounds. The proportion of respondents that ‘agreed’ or ‘strongly agreed’ that their recommendations for patient care are routinely implemented was 69.5% (*n* = 363). Although 51.1% (*n* = 239) ‘agreed’ or ‘strongly agreed’ that they attend multi-disciplinary education sessions (i.e., grand rounds, journal clubs, seminars), 35% (*n* = 164) ‘disagreed’ or ‘strongly disagreed’, and 13.9% (*n* = 65) were neutral. About 84% (*n* = 451) ‘agreed’ or ‘strongly agreed’ that they are encouraged to raise concerns regarding patient care. Respondents that value the role of other healthcare professionals were 98% (*n* = 537), and 62% (*n* = 337) feel that there is a climate of mutual respect within the healthcare team. More than 97% (*n* = 532) respondents value input from members of the healthcare team who are outside their professional role.

### 3.4. Ranking of IPEC Competencies

**Figure 1:**
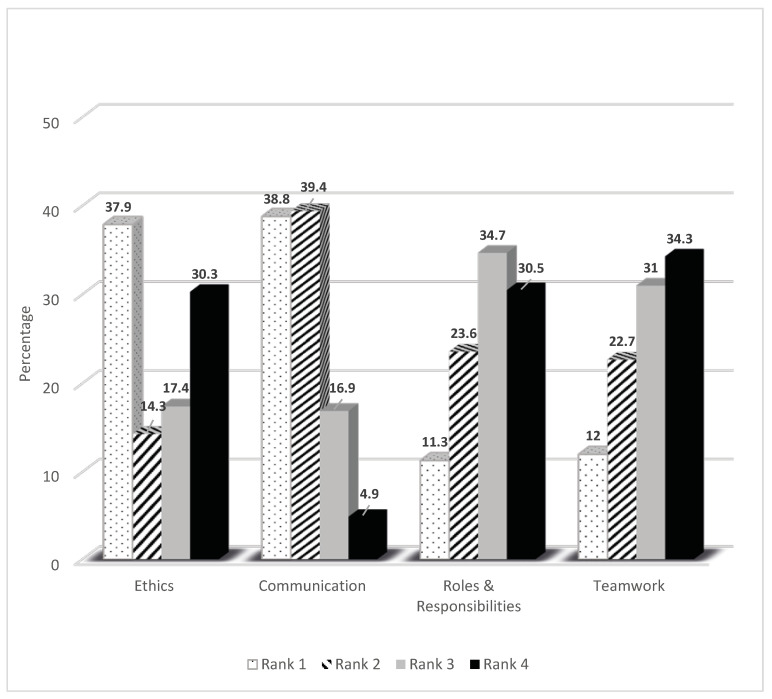
Interprofessional Educational Collaborative (IPEC) Core Competencies Ranking by Healthcare Professionals. *N* = 551.

Figure 1 shows the percentage of responses for each of the competencies. Among the 551 respondents, the majority ranked communication as either first (38.8%) or second (39.4%) in order of importance. Ethics was ranked as first by 37.9% of the respondents and last by 30.3%. Approximately one third of the respondents ranked Teamwork (34.3%) and Roles and Responsibilities (30.5%) as the least important competencies.

## 4. Discussion

The current study examined the attitudes of healthcare professionals working in a large academic medical center toward IPC regarding patient care and the healthcare team. It further explored their behavior and experience of IPC. The healthcare professionals appreciated the importance of IPC in the effective delivery of patient care and valued the need for a collaborative team environment. There was strong consensus among the healthcare professionals that IPC facilitates efficient patient care, improves patient problem solving ability, increases the likelihood of better clinical outcomes for patients, and increases providers’ ability to make optimal patient care decisions. In addition, there was strong agreement generally that IPC does not complicate patient care. Overall, the responses from the healthcare professionals were in support of IPC, and there were no statistically significant differences observed between the grouped professions.

The majority of the healthcare professionals that participated in the current study ranked communication as the IPEC competency of highest importance (ranks 1 and 2). This finding is similar to the results observed in several studies [23,25,26,27]. Healthcare professionals believe that enhanced communication improves teamwork and facilitates cohesive team performance, which in turn ensures safe and optimal care for the patients [26]. Conversely, the current results show that roles and responsibilities were ranked as the competency of least importance, similar to the results seen from a recent study [23]. The low ranking of roles and responsibilities by healthcare professionals may not suggest that the role of other patient care providers is not valued, since 98% of the respondents acknowledged that they value the role of other healthcare professionals. Instead, this may imply that roles and responsibilities are not considered to be of high importance when compared to the other IPEC competences such as ethics and communication. This may also explain the reason for the low ranking of teamwork. This observation warrants that healthcare administrators provide training targeted at creating awareness of the importance of each IPEC competence among healthcare providers during initial hiring, and on a regular basis throughout the duration of employment. A further step would be to promote the introduction of Interprofessional Education (IPE) within the medical and healthcare educational curriculum in order to build the foundation for increasing the appreciation of teamwork and of the roles performed by various healthcare professionals.

The varying levels of consensus about the importance and ranking of IPEC competencies may result from conflicting perceptions among the professionals. Some professions may not concede as much as other professions the importance of IPC and the ranking of the four IPEC competencies. For instance, although the majority of respondents for this study ‘disagreed’ or ‘strongly disagreed’ that working in an interprofessional manner unnecessarily complicates patient care, about 22% ‘agreed’ or ‘strongly agreed’. Also, ethics was ranked as first by 38% of the respondents and last by 30%. A similar study of attitudes toward collaboration of nurses, general practitioners, and hospitalists practicing in newly established Medical Homes in Italy revealed that nurses reflected a significantly more positive attitude toward collaboration compared with general practitioners and hospitalists [28]. Another reason for the differences in the ranking of IPEC competencies may be attributed to receiving IPE. Although the concept of IPE has existed for many years, several educational institutions started incorporating IPE into their curricula during the last decade. Our data reveal that 48% of respondents who were less than 50 years old received IPE in their training curricula, compared to 23% of respondents that were older than 50 years. Also 50% of the respondents who have practiced for less than 15 years in their profession received IPE in their training curricula, compared to 33% with over 15 years of professional practice. These conflicting perceptions of professional roles may impede a successful transition to integrated healthcare.

This study revealed that overall more than a third (35%) of the respondents did not attend multidisciplinary education sessions (grand rounds, seminars, etc.), and more than one-fifth (23%) did not participate in bedside patient care rounds. Most of the respondents that did not attend multidisciplinary education sessions worked in the Neonatal Intensive Care Unit (49%), the adult Emergency Department (41%), the pediatric Emergency Department (29%), and the adult In-patient Department (29%) of the institution. Most of the respondents that did not attend bedside patient care rounds worked in the adult Emergency (34%) and the adult In-patient Departments (28%). The reason may be that these healthcare professionals work late shifts or have schedules that do not allow them to interact physically with caregivers from other professions. Institutional policies that facilitate interprofessional learning and interactions for this group of healthcare professionals should be formulated. Online distant learning and interaction and simulation-enhanced interprofessional education are options for addressing this barrier.

The major limitations of this study are self-reporting and personal views. These are characteristic of the subjective nature of survey research. In addition, the study was confined to one medical center only, and the results therefore may not be generalizable to reflect the entire community of healthcare professionals nationally. The small survey response rate of about 16% is another limitation. This response rate is similar to the response rates reported utilizing internet based surveys. A systematic review of internet-based surveys of health professionals showed that the response rate varied between 9% and 94%, depending on the number of email reminders provided [29]. Exposure of healthcare professionals to job strain may be an important influence on survey response [30], and workers who are not compensated for their time in completing a survey may account for low response rates.

## 5. Conclusions

Our evaluation of the attitudes of healthcare professionals from several disciplines toward IPC regarding patient care and the healthcare team, and their behavior and experience of IPC revealed that healthcare professionals believe that communication among team members, understanding of role limitations, and trust and respect among team members of different disciplines are necessary for IPC to be successful. Effective collaboration provides an environment for teamwork that promotes safe and improved patient-centered care. It is encouraging to observe from the results of this study, the strong support for IPC among healthcare professionals. This may present more opportunities for collaborative initiatives and the optimization of patient care in the academic medical center under investigation. Hospital administrators should regularly assess and foster the four IPEC competencies with the goal of building and maintaining collaborative environments where health professionals can provide high quality integrated patient care. Further research is needed to evaluate the components of IPC such as communication and coordination in the academic medical center under the current investigation, and to assess the perspectives of hospital administrators regarding IPC and IPE.

## Figures and Tables

**Table 1:** Characteristics of the study participants.

	Healthcare Professions						
Variables	Total	Advanced Nurse Practitioners	Physicians (MD, Fellows, Residents)	Nursing	Allied Health (RD, PA, PT, OT, SLP, SW, RT)	Pharmacist	Other Professional
	*N* = 551 (%)	*N* = 15 (%)	*N* = 138 (%)	*N* = 316 (%)	*N* = 65 (%)	*N* = 14 (%)	*N* = 3 (%)
Gender							
Males	126 (22.9)	1 (6.7)	87 (63.0)	23 (7.3)	10 (15.4)	4 (28.6)	1 (33.3)
Females	425 (77.1)	14 (93.3)	51 (37.0)	293 (92.7)	55 (84.6)	10 (71.4)	2 (66.7)
Age in Years							
20–29	76 (13.8)	2 (13.3)	25 (18.1)	26 (8.2)	17 (26.2)	6 (42.9)	0 (0)
30–39	135 (24.5)	3 (20.0)	51 (37.0)	63 (19.9)	15 (23.1)	3 (21.4)	0 (0)
40–49	138 (25.0)	7 (46.7)	20 (14.5)	91 (28.8)	15 (23.1)	3 (21.4)	2 (66.7)
50–59	140 (25.4)	1 (6.7)	23 (16.7)	100 (31.6)	16 (24.6)	0 (0)	0 (0)
60–69	54 (9.8)	2 (13.3)	14 (10.1)	33 (10.4)	2 (3.1)	2 (14.3)	1 (33.3)
>69	8 (1.5)	0 (0)	5 (3.6)	3 (0.9)	0 (0)	0 (0)	0 (0)
Ethnic Background							
White, non-Hispanic	408 (74.0)	12 (80)	93 (67.4)	237 (75.0)	52 (80.0)	12 (85.7)	2 (66.7)
Black, non-Hispanic	68 (12.3)	2 (13.3)	11 (8.0)	48 (15.2)	7 (10.8)	0 (0)	0 (0)
Hispanic	16 (2.9)	0 (0)	13 (9.4)	3 (0.9)	0 (0)	0 (0)	0 (0)
Asian/non-Pacific Islander, non-Hispanic	34 (6.2)	0 (0)	16 (11.6)	13 (4.1)	3 (4.6)	1 (7.1)	1 (33.3)
Multiple races	20 (3.6)	1 (6.7)	3 (2.2)	13 (4.1)	2 (3.1)	1 (7.1)	0 (0)
Other	5 (0.9)	0 (0)	2 (1.4)	2 (0.6)	1 (1.5)	0 (0)	0 (0)
Highest Degree							
Associates	104 (18.9)	0 (0)	0 (0)	96 (30.4)	8 (12.3)	0 (0)	0 (0)
Bachelors	198 (35.9)	0 (0)	0 (0)	171 (54.1)	25 (38.5)	1 (7.1)	1 (33.3)
Masters	76 (13.8)	11 (73.3)	0 (0)	40 (12.7)	24 (36.9)	1 (7.1)	0 (0)
Doctoral	30 (5.4)	4 (26.7)	2 (1.4)	2 (0.6)	8 (12.3)	12 (85.7)	2 (66.7)
Medical	136 (24.7)	0 (0)	136 (98.6)	0 (0)	0 (0)	0 (0)	0 (0)
No Response	7 (1.3)	0 (0)	0 (0)	7 (2.2)	0 (0)	0 (0)	0 (0)
Years of Practice							
<5	84 (15.2)	0 (0)	37 (26.8)	26 (8.2)	16 (24.6)	5 (35.7)	0 (0)
5–10	97 (17.6)	3 (20.0)	32 (23.2)	44 (13.9)	14 (21.5)	4 (28.6)	0 (0)
11–15	66 (12.0)	4 (26.7)	15 (10.9)	38 (12.0)	7 (10.8)	1 (7.1)	1 (33.3)
16–20	69 (12.5)	1 (6.7)	11 (8.0)	45 (14.2)	10 (15.4)	1 (7.1)	1 (33.3)
>20	235 (42.6)	7 (46.7)	43 (31.2)	163 (51.6)	18 (27.7)	3 (21.4)	1 (33.3)
Area of work							
Inpatient	291 (52.8)	9 (60.0)	67 (48.6)	164 (51.9)	45 (69.2)	6 (42.9)	0 (0)
Outpatient	144 (26.1)	4 (26.7)	44 (31.9)	74 (23.4)	16 (24.6)	6 (42.9)	0 (0)
Emergency	36 (6.5)	1 (6.7)	16 (11.6)	19 (6.0)	0 (0)	0 (0)	0 (0)
Other Locations	80 (14.5)	1 (6.7)	11 (8.0)	59 (18.7)	4 (6.2)	2 (14.3)	3 (100.0)

**Table 2:** Healthcare Professionals’ Attitudes toward Patient Care and the Healthcare Team.

**A: Attitudes Toward Interprofessional Collaboration Regarding Patient Care**						
**Questions**	**Responses (*N* = 551)**					
	**Strongly Disagree *N* (%)**	**Disagree *N* (%)**	**Neither Agree nor Disagree *N* (%)**	**Agree *N* (%)**	**Strongly Agree *N* (%)**	**Mean ± SD**
Patients receiving interprofessional care are more likely than others to be treated as a whole person	3 (0.5)	14 (2.5)	41 (7.4)	202 (36.7)	291 (52.8)	4.39 ± 0.78
Patients would ultimately benefit if health care professionals worked together to solve patient problems	1 (0.2)	1 (0.2)	3 (0.5)	120 (21.8)	426 (77.3)	4.76 ± 0.48
The “give and take” among team members helps providers make better patient care decisions	2 (0.4)	10 (1.8)	43 (7.8)	231 (41.9)	265 (48.1)	4.36 ± 0.73
The interprofessional approach makes the delivery of patient care more efficient	4 (0.7)	11 (2.0)	59 (10.7)	176 (31.9)	301 (54.6)	4.38 ± 0.81
Reporting observations to a multidisciplinary team helps team members better understand the role of other health care professionals	1 (0.2)	7 (1.3)	34 (6.2)	197 (35.8)	312 (56.6)	4.47 ± 0.69
Interprofessional collaboration increases the health care teams ability to understand clinical problems	1 (0.2)	1 (0.2)	26 (4.7)	206 (37.4)	317 (57.5)	4.52 ± 0.61
Working in an interprofessional manner unnecessarily complicates patient care	157 (28.5)	212 (38.5)	59 (10.7)	59 (10.7)	64 (11.6)	2.38 ± 1.31
**B: Attitudes Toward Interprofessional Collaboration regarding the Healthcare Team**						
**Questions**	**Responses (*N* = 551)**					
Interprofessional collaboration helps healthcare professionals think positively about the health care team	2 (0.4)	9 (1.6)	82 (14.9)	255 (46.3)	203 (36.8)	4.18 ± 0.77
Communication skills are critical for the healthcare team for improved patient outcomes	0 (0)	1 (0.2)	8 (1.5)	121 (22.0)	421 (76.4)	4.75 ± 0.48
Interprofessional collaboration allows healthcare professionals to understand their role limitations	2 (0.4)	24 (4.4)	93 (16.9)	248 (45.0)	184 (33.4)	4.07 ± 0.84
For interprofessional collaboration to be effective, the healthcare team needs to trust and respect each other	2 (0.4)	0 (0)	9 (1.6)	148 (26.9)	392 (71.1)	4.68 ± 0.54
Team meetings foster communication among members from different professions and disciplines	0 (0)	6 (1.1)	42 (7.6)	234 (42.5)	269 (48.8)	4.39 ± 0.68
Working in an interprofessional environment keeps health professionals enthusiastic and interested in their jobs	3 (0.5)	14 (2.5)	124 (22.5)	228 (41.4)	182 (33.0)	4.04 ± 0.84
Working in an interprofessional manner requires additional time	18 (3.3)	89 (16.2)	143 (26.0)	196 (35.6)	105 (19.1)	3.51 ± 1.07
For interprofessional collaboration to be effective, members of the healthcare team must work within their scope of practice	0 (0)	12 (2.2)	52 (9.4)	258 (46.8)	229 (41.6)	4.28 ± 0.72

**Table 3:** Healthcare Professionals’ Behavior and Experience Regarding Interprofessional Collaboration.

Questions	Responses						
	*N* *	Strongly Disagree *N* (%)	Disagree *N* (%)	Neither Agree nor Disagree *N* (%)	Agree *N* (%)	Strongly Agree *N* (%)	Mean ± SD
I am included in patient care decision making	533	11 (2.1)	34 (6.4)	55 (10.3)	236 (44.3)	197 (37.0)	4.08 ± 0.95
I participate in multi-disciplinary patient care rounds	464	25 (5.4)	80 (17.2)	62 (13.4)	166 (35.8)	131 (28.2)	3.64 ± 1.21
My recommendations for patient care are routinely implemented	525	17 (3.2)	36 (6.9)	107 (20.4)	242 (46.1)	123 (23.4)	3.80 ± 0.98
I attend multi-disciplinary education sessions (i.e., grand rounds, journal clubs, seminars)	468	47 (10.0)	117 (25.0)	65 (13.9)	133 (28.4)	106 (22.7)	3.29 ± 1.33
I am encouraged to raise concerns regarding patient care	540	14 (2.6)	25 (4.6)	50 (9.3)	247 (45.7)	204 (37.8)	4.11 ± 0.94
I value the role of other healthcare professionals	548	1 (0.2)	1 (0.2)	9 (1.6)	177 (32.3)	360 (65.7)	4.63 ± 0.55
I feel there is a climate of mutual respect within the healthcare team	543	31 (5.7)	82 (15.1)	93 (17.1)	232 (42.7)	105 (19.3)	3.55 ± 1.13
I value input from members of the healthcare team who are outside my own professional role	546	1 (0.2)	3 (0.5)	10 (1.8)	232 (42.5)	300 (54.9)	4.51 ± 0.58

Note: * Responses do not total *n* = 551 due to exclusion of ‘Do not apply’ and missing data.
